# Prednisolone Delivery Platforms: Capsules and Beads Combination for a Right Timing Therapy

**DOI:** 10.1371/journal.pone.0160266

**Published:** 2016-07-29

**Authors:** Andrea Cerciello, Giulia Auriemma, Silvana Morello, Rita P. Aquino, Pasquale Del Gaudio, Paola Russo

**Affiliations:** 1 Department of Pharmacy, University of Salerno, Fisciano, Italy; 2 PhD Program in Drug Discovery and Development, University of Salerno, Fisciano, Italy; Duke University Marine Laboratory, UNITED STATES

## Abstract

In this work, a platform of alginate beads loaded with Prednisolone in hypromellose/gellan gum capsules (F6/Cps) able to delay steroidal anti-inflammatory drug (SAID) release as needed for chronotherapy of rheumatoid arthritis is proposed. Rheumatoid arthritis, showing a worsening in symptoms in the morning upon waking, is a pathology that can benefit from chronotherapy. With the aim to maximize prednisolone therapeutic action allowing the right timing of glucocorticoid therapy, different engineered microparticles (gel-beads) were manufactured using prilling (laminar jet break-up) as micro-encapsulation technique and Zn-alginate as gastroresistant carrier. Starting from various feed solutions and process parameters, the effect of the variables on particles size, morphology, solid state properties and drug release was studied. The optimization of operative and prilling/ionotropic gelation variables led to microspheres with almost spherical shape and a narrow dimensional range. The feed solution with the highest alginate (2.5% w/v) amount and drug/polymer ratio (1:5 w/w) gave rise to the highest encapsulation efficiency (78.5%) as in F6 formulation. As to drug release, F6 exhibited an interesting dissolution profile, releasing about 24% of the drug in simulated gastric fluid followed by a more sustained profile in simulated intestinal fluid. #F6, acting as a gastro-resistant and delayed release formulation, was selected for in vivo studies on male Wistar rats by means of a carrageenan-induced oedema model. Finally, this efficacious formulation was used as core material for the development of a final dosage form: F6/Cps allowed to significantly reduce prednisolone release in simulated gastric fluid (12.6%) and delayed drug release up to about 390 minutes.

## Introduction

Pharmaceutical chronotherapy consists in administering drugs at optimal times of the day following circadian rhythms with the primary purpose to increase the therapeutic effectiveness while decreasing systemic side effects of drugs. The so called “early morning pathologies” are among the diseases that may benefit from chronotherapy: in inflammatory arthritis, for example, joint swelling, pain and stiffness are worse in the morning upon waking than during the day and can seriously impair patients quality of life. These morning symptoms are justified by a circadian rhythm of inflammatory mechanisms: compared to healthy subjects, the peak time of pro-inflammatory mediators, mainly interleukin (IL)-6 and tumour necrosis factor (TNF)-α, seems to shift toward the wake-up time, [1–3].

The main goal of a steroidal anti-inflammatory drug (SAID) treatment should be to reduce the early morning symptoms. With this aim, a glucocorticoid in a conventional oral delivery should be administered approximately at 2:00 am [4,5], causing a poor patient compliance or ineffectiveness if the drug is taken upon awakening [6–9]. Prednisolone and prednisone are the most widely used steroidal agents for the oral therapy of chronic inflammation diseases because of their short half-life and relatively low side effects, compared to other SAIDs. The drugs are metabolically interconvertible, prednisolone being the pharmacologically active species. Both drugs are rapidly absorbed after oral administration with plasma half-lives of about 3.5h for prednisone and 2.8h for prednisolone. However, prednisolone bioavailability after the administration of an oral prednisone dose is approximately 80% compared to similar prednisolone dosage form. [10,11]. Moreover, prednisolone administration seems to be preferable also in case of liver disease, because of the poor conversion of prednisone to prednisolone [12].

Despite the number of scientific papers on controlled-release forms of prednisolone [13–16], there are currently no products available on the market following a chronotherapeutic approach of the early morning pathologies.

In this work based on prednisolone (P) properties, a platform of alginate beads loaded with P in hypromellose/gellan gum capsules (F6/Cps) is proposed to delay SAID release as needed for chronotherapy of rheumatoid arthritis. In a first step, engineered particles were produced by prilling/ionotropic gelation technique using alginate as gastroresistant carrier and Zinc as crosslinking agent. The main critical process variables such as characteristics of biopolymers and composition of the prilling feed solutions, composition and pH of the aqueous bulks for ionotropic gelation of the polymer/drug drops and cross-linking time were examined as well as their effect on particles size, morphology and drug release profiles. The formulation showing suitable gastro-resistant properties and a delayed release of the drug was selected for *in vivo* experiments on male Wistar rats, by a carrageenan-induced oedema model. Finally, hypromellose/gellan gum based capsules (DRcaps^®^) were used as carrier system of these particles to obtain a final dosage form combining the efficacy and the delayed release properties of the beads and the ability of hypromellose/gellan gum to slow down capsule opening after swallowing.

## Materials and Methods

### Materials

Sodium alginate (European Pharmacopoeia X, MW ≈ 240 KDa) was purchased from Carlo Erba (Milan, Italy); prednisolone (P) and zinc acetate dihydrate (Zn(CH_3_COO)_2_·2H_2_O) were supplied by Sigma–Aldrich (Milan, Italy). DR^®^ capsules size 3 were kindly donated by Capsugel (Milan Italy). All other chemicals and reagents were obtained from Sigma Aldrich and used as provided.

### Methods

#### Drug loaded beads manufacture

Different amounts of sodium alginate were dissolved in deionized water at room temperature under gentle stirring in order to obtain three feed solutions with different polymer concentration (2.00%, 2.25% and 2.50% w/w). Afterwards, for each concentration, different amounts of prednisolone were added to the polymeric solution and stirred for 4 h in order to obtain two different drug/polymer ratios (1:10 and 1:5 w/w) and six different formulations, consequently. Beads were produced by a vibrating nozzle device (Nisco Encapsulator Unit, Var D; Nisco Engineering Inc., Zurich, CH), equipped with a syringe pump (Model 200 Series, Kd Scientific Inc., Boston, MA, USA), pumping the drug/polymer solution through a nozzle 600 μm in diameter [17]. Volumetric flow rate was fixed at 5 ml/min; vibration frequency was set at 350 Hz, with amplitude of vibration 100%. The distance between the vibrating nozzle and the gelling bath was maintained at 25 cm. The droplets obtained were collected into an aqueous solution of Zn(CH_3_COO)_2_·2H_2_O (10% w/v, pH = 1.5) where they were gelled under gentle stirring for 2 min and then washed with deionized water. In the end, beads were dried at room temperature by exposure to air (22°C; 67% RH) for several hours (12–18 h) until constant weight was reached.

#### Drug content and encapsulation efficiency

In order to quantify P content, Zn-alginate matrix were broken up adding to each formulations (about 10 mg of dried beads) to 2 ml of phosphate buffer (100 mM, pH 7.0) and keeping them under vigorous stirring for 24h. Successively, 23 ml of ethanol were added and the suspension was centrifuged at 6000 rpm for 10 min. P content was evaluated by spectrophotometric analysis at a λ of 244 nm (Evolution 201 UV/VIS Spectrometer, Thermo Scientific, Waltham; MA, USA). The actual drug content (ADC) was obtained [18], as reported below:

$$ADC(\%)=\frac{drug\;content\;in\;dry\;beads}{weight\;of\;dry\;beads}\times 100$$ Eq 1

Finally, encapsulation efficiency was calculated as the ratio of actual to theoretical drug content, i.e. the weight of drug added (g)/weight of polymers and drug added (g) x 100. Each experiment was performed in triplicate, and results were expressed as mean ± standard deviation.

#### Beads morphology and swelling behaviour

With the aim to evaluate size distribution and morphology of produced beads, Scanning Electron Microscopy (SEM), was employed using a Carl Zeiss EVO MA 10 microscope with a secondary electron detector (Carl Zeiss SMT Ltd., Cambridge, UK) equipped with a LEICA EMSCD005 metallizer producing a deposition of a 200–400°A thick gold layer. Analyses were conducted at 17 keV. Projection diameter was appreciated by image analysis (Image J software, Wayne Rasband, National Institute of Health, Bethesda, MD, USA). A minimum of one hundred beads microphotographs were studied for each formulation and relative standard deviation for at least three different prilling processes was evaluated. Sphericity Coefficient (SC) was calculated as reported in Eq 2 [19,20], using perimeter (p) and projection surface area (A) data obtained by image analysis [21]:

$$SC=\frac{4\pi A}{p^{2}}$$ Eq 2

Beads inner matrix was studied by SEM images of cryofractured beads. These latter were prepared by breaking with two needles frozen particles obtained by immersion into liquid nitrogen for 60 s.

Swelling behaviour of the beads was evaluated by optical microscopy. A Leica inverted microscope equipped with a CCD camera (Leica, Milan, Italy) was employed. The beads were immersed in gastric simulated fluid for two hours and in intestinal simulated fluid until complete dissolution and observed at established time points. Swelling degree was determined as follow:

$$\%swelling=\frac{bead\;size\;at\;time\;t-bead\;size\;at\;time\;zero}{bead\;size\;at\;time\;zero}∙100$$ Eq 3

All measurements were done in triplicate and the variability expressed as S.D.

#### Calorimetric analyses

Thermal characteristics of P loaded microparticles were studied by differential scanning calorimetry (DSC) (Mettler Toledo DSC 822e module controlled by Mettler Star E software, Columbus, OH, USA), and compared to both blank beads and prednisolone raw material thermal profiles. About 25–30 mg of sample were placed in a standard aluminium crucible that was pierced and heated from 25°C to 350°C with a heating rate of 20°C/min in nitrogen atmosphere at a flow rate of 150 ml/min. Distinctive peaks were recorded and specific melting temperature was evaluated. Analyses were conducted in triplicate.

#### Fourier Transform Infrared Spectroscopy (FT-IR) analyses

FT-IR spectra were detected as KBr powder dispersion (Jasco model FT/IR-410, 420 Herschel series–Jasco Corporation Tokyo, Japan) using the EasiDiffTM Diffuse Reflectance Accessory. The samples were combined with small amount of potassium bromide and pressed to 6 tons in a manual press (OMCN s.p.a., Bergamo, Italy). The thin compacts produced were analysed using 256 scans and with a 1 cm^−1^ resolution step. Each experiment was carried in triplicate and results averaged.

#### Drug release studies

*In vitro* dissolution experiments for each formulation compared to prednisolone raw material were conducted in sink conditions using a *United State Pharmacopoeia* (USP) 36 dissolution apparatus II (paddle at 75 rpm, temperature at 37°C) (Sotax AT7 Smart–Sotax, CH). Samples were added to 750 ml of 0.1 M HCl for 2h, then 250 ml of 0.2 M Na_3_PO_4_ were added and pH adjusted to 6.8 as expected from <1092> monograph “The Dissolution Procedure: Development and Validation” (USP 36). Prednisolone quantification was performed spectrophotometrically at a λ of 244 nm. All experiments were performed on six different batches, reporting data as mean values ± standard deviations. Finally, formulations showing the best results in terms of EE and drug dissolution profile were introduced into selected capsules (DR^®^ caps) and P release from these dosage forms was evaluated using the USP-II apparatus equipped with mesh basket (USP Standard Size Mesh, 40) and the experimental protocol previously reported.

#### Animals and carrageenan oedema induction

Male Wistar rats (180–220g) were purchased from Envigo (Envigo RMS—S.r.l., Italy). Rats were anaesthetized with isoflurane and oedema was induced by injecting in the right hind paw 100μl of carrageenan 1% (w/v) as previously reported [22]. Paw volume was measured plethysmographycally (2Biological-Instruments, Italy) at the time zero (0.5 h), each hour for 6 h, and at 24h after carrageenan injection. Data were expressed as mean ± SEM. Statistical differences were evaluated by two-way analysis of variance (ANOVA) followed by Bonferroni’s post test. P value of <0.05 was considered statistically significant.

All the experiments were approved by Italian Health Ministry and conducted according to institutional animal care guidelines, Italian Law 26/2014 based on the European Community Law for Animal Care 2010/63/UE.

#### In vivo experiments

To assess the *in vivo* anti-inflammatory activity of the optimized formulation F6 in comparison with the pure drug, rats were orally treated by gavage with F6 or pure drug in 0.5% (w/v) methyl cellulose (MC) [23] at a prednisolone equivalent dose of 3 mg kg^-1^; this dose was selected in preliminary studies as it achieved optimal anti-inflammatory effects. Control groups received 0.5 mL of MC [24] for drug administration experiments or blank Zn-alginate beads (F) in 0.5% (w/v) MC for beads administration experiments, simultaneously with the injection of the phlogistic agent. Prednisolone raw material was administered 0.5h, 5h and 15h before the phlogistic injury, while beads #F6 were administered to rats 5h or 15h before the carrageenan injection, in order to verify their effectiveness in control drug release.

## Results and Discussion

### Beads production and characterization

P loaded hydrophilic microparticles (gel-beads) were manufactured using prilling (laminar jet break-up) as mild and easy scalable micro-encapsulation technique. The main critical variables of the prilling and gelling process (cross-linking divalent cations; pH of the gelling solution and cross-linking time) were studied and optimized according to prednisolone physico chemical properties [25,26].

Six formulations (#F1 –#F6) were obtained, varying alginate amounts (from 2.0% to 2.5% w/v) and drug/polymer mass ratios (1:10 and 1:5 w/w), with very short processing time in the selected operative conditions (Table 1). As control, blank Zn-alginate beads were also produced (F). Drying process was conducted by exposing hydrated beads to standard room conditions (22°C; 67% RH) for 12–18 h until constant weight was reached.

**Table 1:** Composition, actual drug content, encapsulation efficiency, mean diameter and sphericity coefficient of optimized manufactured by prilling.

Formulation code	Alginate concentration (%w/v)	P/Alg mass ratio (w/w)	ADC (% ± SD)	EE (% ± SD)	Dried beads diameter (mm ± SD)	SC
**F**	2.50	-	-	-	2.4 ± 0.15	0.93 ± 0.01
**F1**	2.00	1:10	6.2 ± 0.2	67.9 ± 2.0	2.4 ± 0.12	0.91 ± 0.02
**F2**	2.00	1:5	11.7 ± 0.6	69.9 ± 3.8	2.7 ± 0.13	0.90 ± 0.02
**F3**	2.25	1:10	6.6 ± 0.3	72.8 ± 3.4	2.4 ± 0.12	0.90 ± 0.01
**F4**	2.25	1:5	12.3 ± 0.4	73.6 ± 2.3	2.5 ± 0.12	0.91 ± 0.02
**F5**	2.50	1:10	6.8 ± 0.2	74.3 ± 2.0	2.4 ± 0.12	0.92 ± 0.02
**F6**	2.50	1:5	13.1 ± 0.3	78.6 ± 2.1	2.5 ± 0.12	0.90 ± 0.02

Actual drug content (ADC) and encapsulation efficiency (EE) values of all dried formulations ranged from 67.9 to 78.6% (Table 1) in relation to the amount (from 2 to 2.5%) of alginate used as well as to the drug/polymer ratio (from 1:10 to 1:5) selected.

In fact, the highest ADC and EE values were obtained raising drug/polymer ratio and alginate amount into the feed solutions. These data were consistent with previous observations suggesting that the loading of higher amount of drug and an increased polymer concentration may promote, during the gelation phase, intermolecular interactions able to stabilize the “egg-box” structure [27], reducing the leaching of the drug from the droplets into the gelling medium. Accordingly, #F6 (alg. 2.5%, drug/polymer ratio 1:5) showed the best P entrapment within the matrix (EE = 78.6%).

**Fig 1:**
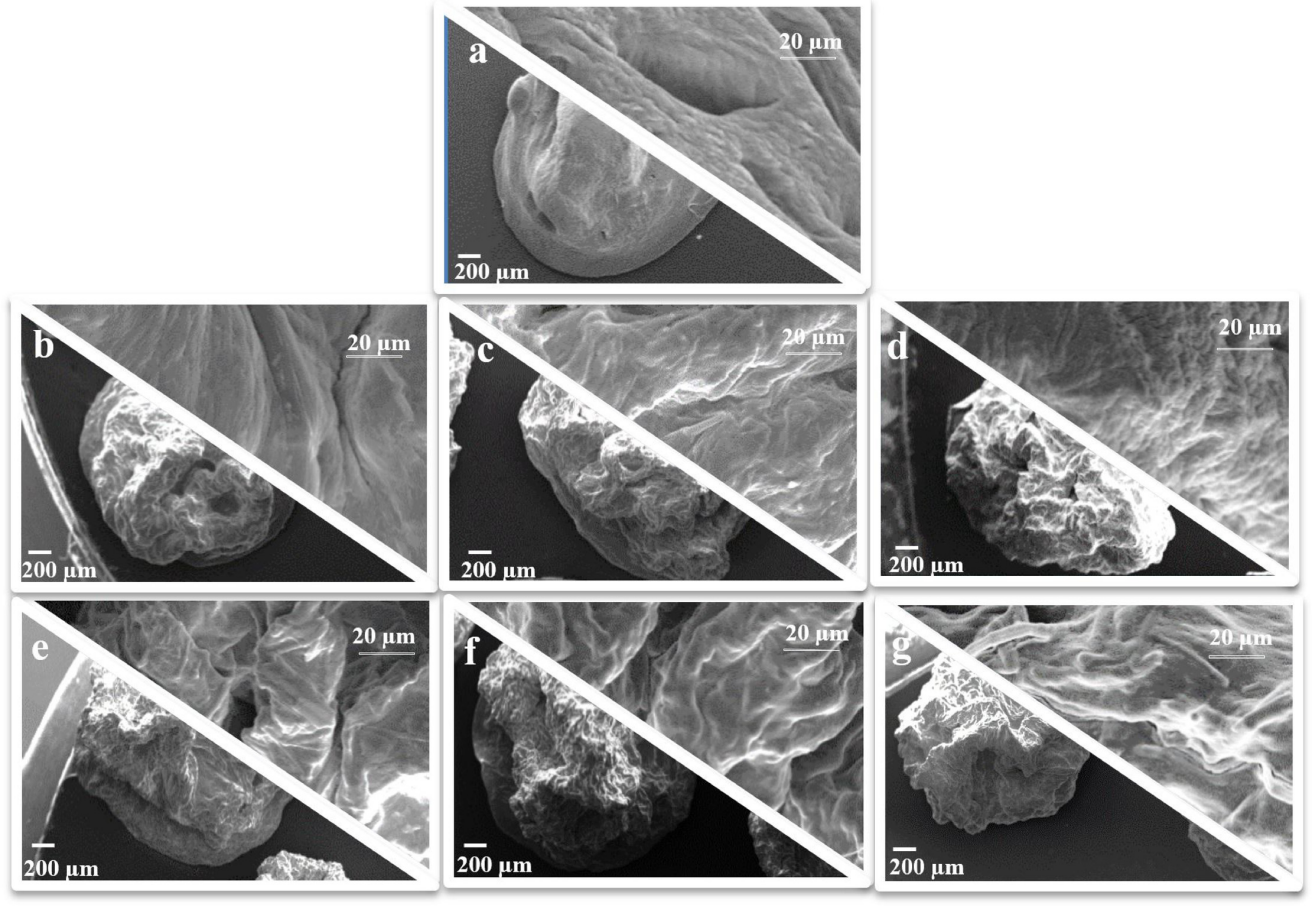
**SEM microphotographs at different magnification of dried zinc-Alg beads:** a) #F, b) #F1, c) #F2, d) #F3, e) #F4, f) #F5, g) #F6.

Polymer and drug concentration in the processed feeds influenced the surface properties of the particles: drug-free beads (#F, Fig 1A) were smoother without wrinkles, whereas P loaded beads had all an irregular surface and roughness directly related to P and polymer content. In particular, the higher the content of drug and the polymer concentration, the greater the surface roughness. Moreover, no drug crystals were observed on the particle surface (#F1-F6, Fig 1B–1G) and all particles showed similar morphological characteristics in terms of sphericity coefficient (SC 0.90–0.92) and mean diameter values (2.4–2.7 mm) (Table 1).

These data suggested that the optimization of operative and process variables during prilling/ionotropic gelation process (Zn^2+^concentration, pH, temperature and curing time) was able to produce microspheres with almost spherical shape in a narrow dimensional range, independently from the amount of alginate or to the drug/polymer ratio selected.

**Fig 2:**
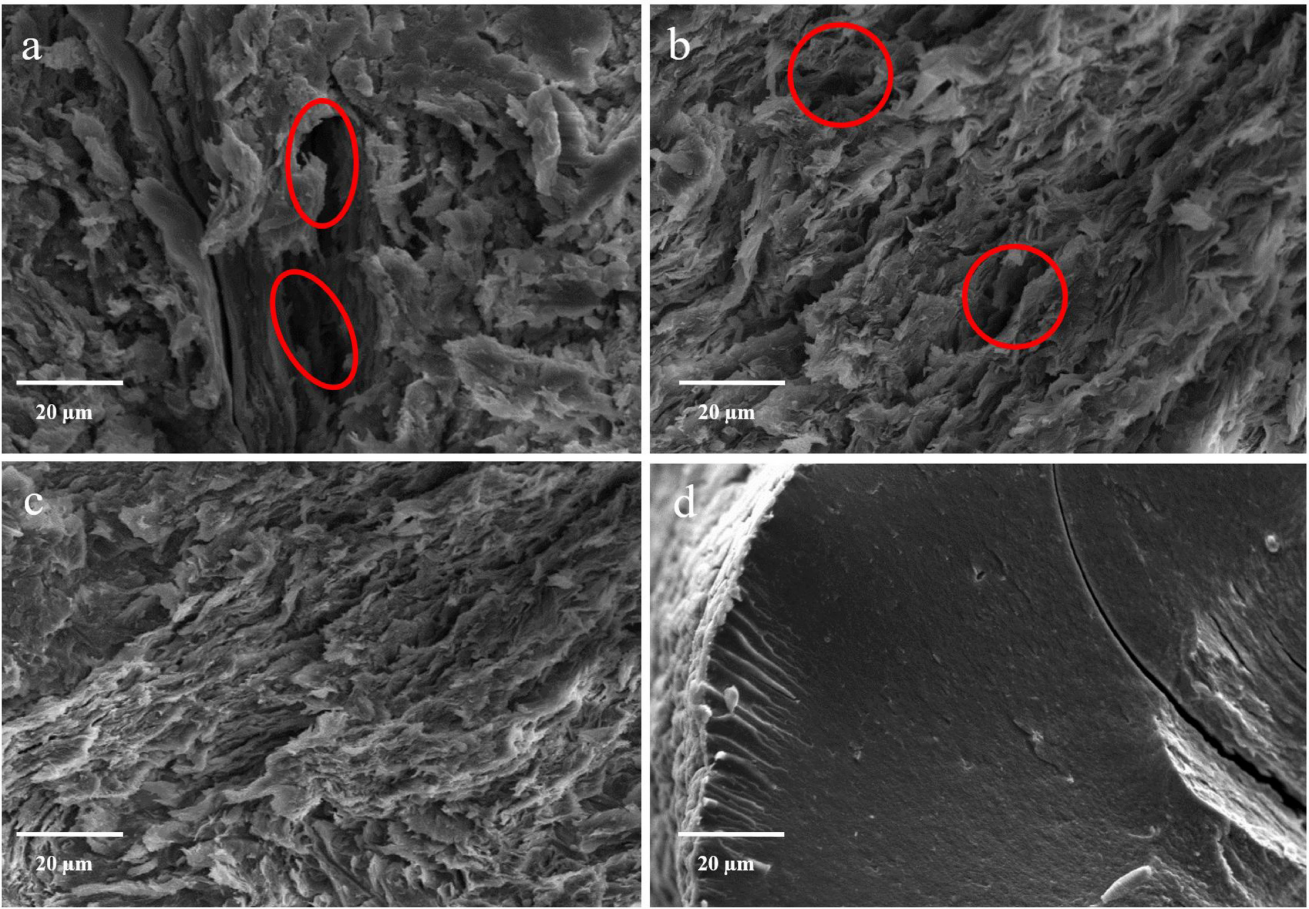
**SEM microphotographs of cryofractured zinc-Alg beads:** #F4 (a), #F5 (b) #F6 (c) and #F (d).

To better understand the influence of drug content and alginate amount on the inner structure, beads were cryofractured prior to SEM observation. The blank Zn-alginate beads revealed a compact inner matrix (#F Fig 2D). On the contrary, the internal structure of all engineered particles was less continuous and interrupted by several empty folders (Fig 2A and 2B, red circles), depending on the polymer and drug concentration into the liquid feed processed. The increase of alginate (from 2.25% to 2.50%, Fig 2A and 2C) and drug concentrations (from 1:10 w/w to 1:5 w/w, Fig 2C) induces a higher organization of the matrix, desirable for the development of oral controlled drug delivery systems.

In order to assess the matrix resistance in biological fluids, swelling experiments were conducted in SGF and SIF, following pH change methods steps. Formulation F6 exhibited in SGF a swelling degree of about 13% after 60 min and 58% after 120 min. Beads presented at both time points smoother surface and higher particle sphericity compared to dried paticles. Interestingly, drug loaded beads exhibited the maximum swelling degree (76%) at 135 min, as a result of enhanced water penetration and and erosion process in SIF. After this time point, the erosion overtakes the swelling, leading to a complete particle disintegration at 190 min.

### DSC and IR analysis

**Fig 3:**
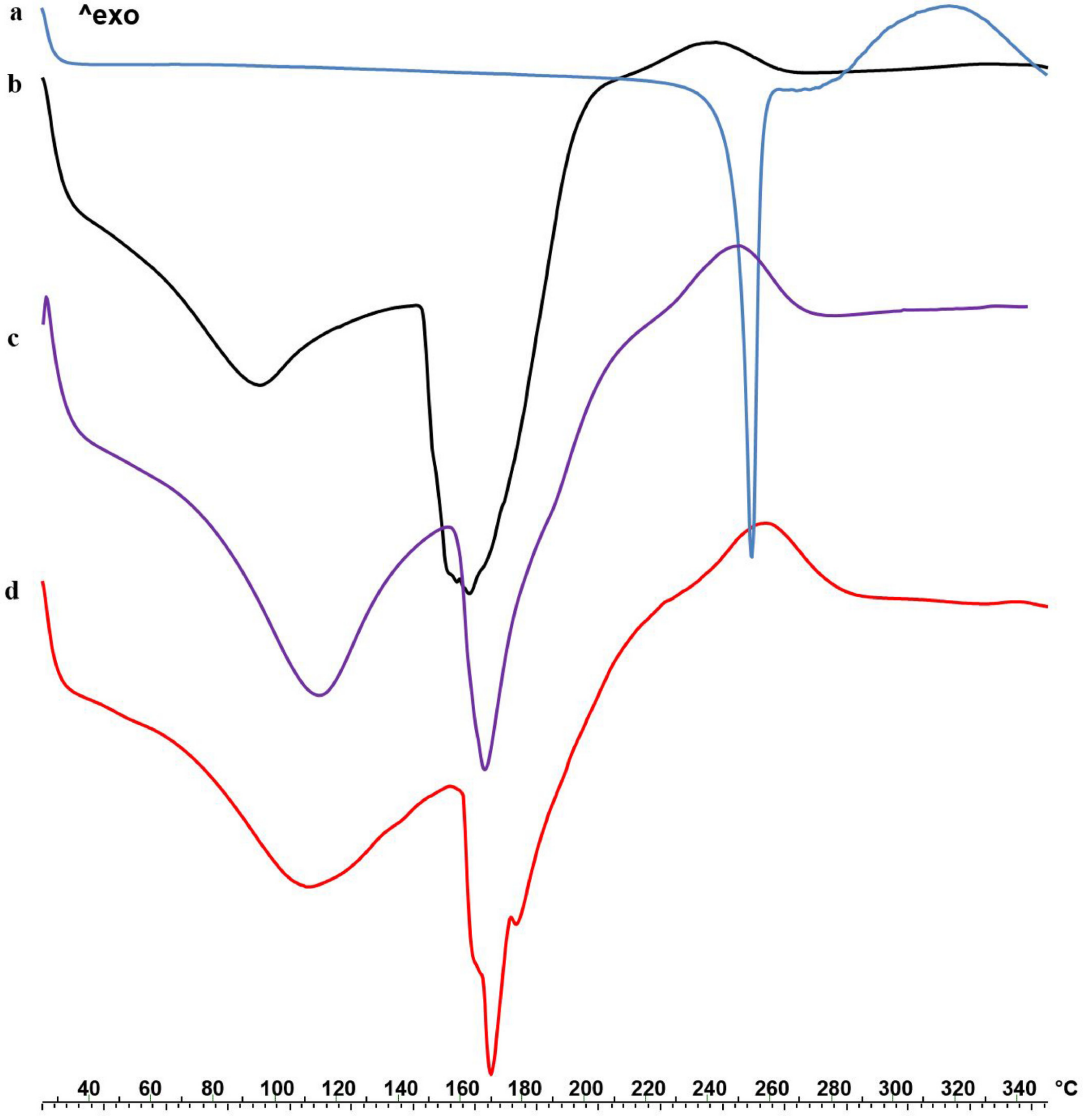
**DSC thermograms of P ramaterial (a), alginate blank beads F (b) and drug loaded formulations F5 (c) and F6 (d)**.

In order to highlight feasible drug-polymer interaction after prilling process, thermal profiles of P raw material, blank Zn-alginate beads and drug loaded beads were studied by differential scanning calorimetry and reported in Fig 3.

P raw material exhibited a clear endothermic peak at 252˚C followed by an exothermic event between 280 and 350 ˚C corresponding to its oxidative degradation. Both empty and P loaded formulations exhibited an opening endothermic event between 90 and 130 ˚C related to the loss of water adsorbed on the particle surface and entrapped into the matrix. Blank Zn-alginate beads, showed a broad endothermic signal between 150 and 182˚C followed by a ramp like event up to 260 ˚C. Interestingly, the endothermic peak due to the fusion of crystalline P (252 ˚C) disappeared in drug loaded beads thermograms (Fig 3C and 3D); moreover, melting as well as degradation peaks were shifted to higher temperatures in F5 and F6 thermal profiles with respect to blank Zn-alginate beads. These effects, more pronounced in F6, suggested a physical alginate/P interaction as effect of drug loading in the polymeric matrix [28].

**Fig 4:**
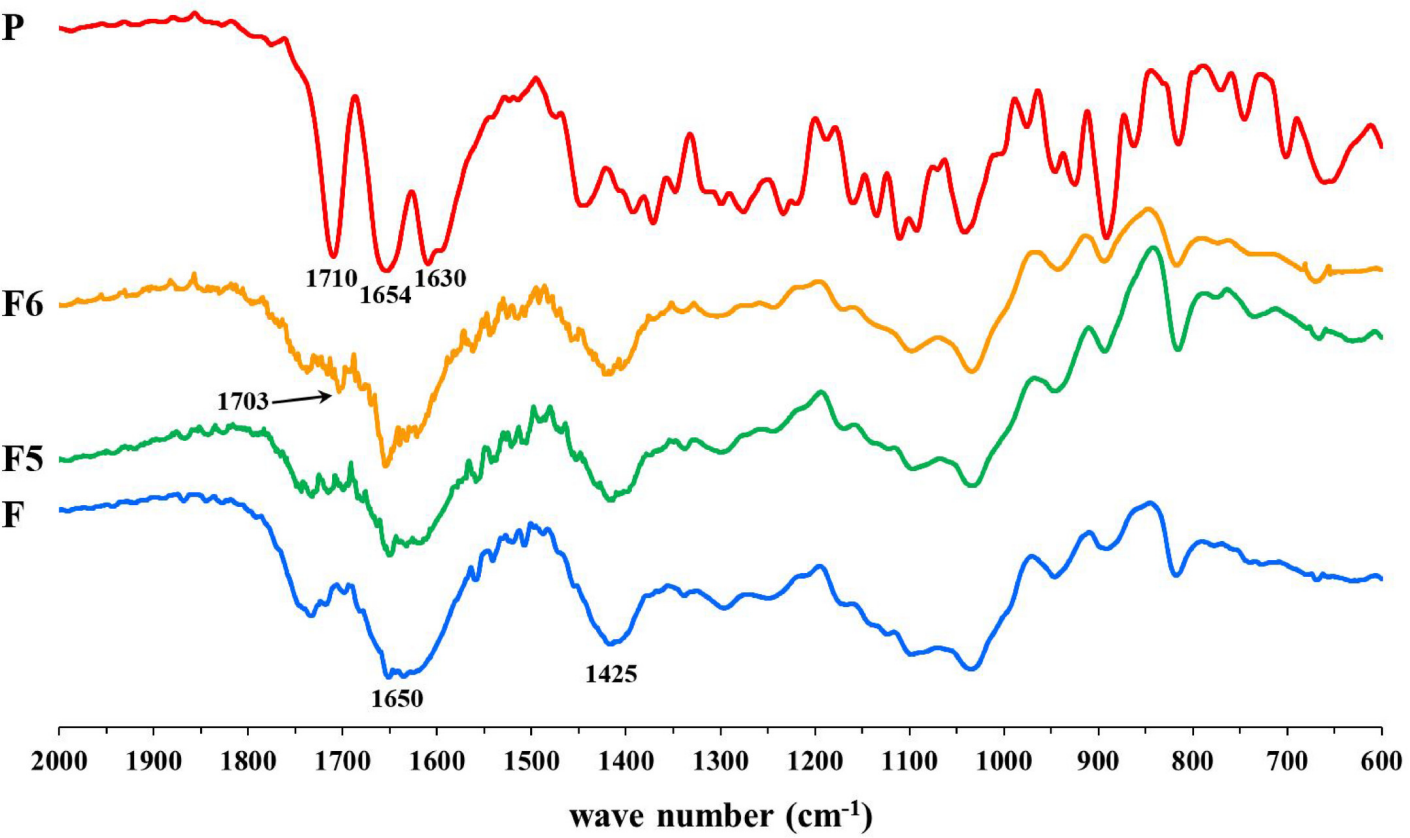
FTIR spectra of P raw material, formulations F5-F6 and Zn-alginate blank beads F.

In order to verify the presence of such interaction suggested by DSC analysis, FT-IR studies were performed and results reported in Fig 4.

P raw material exhibited two characteristic bands in the range 1590–1710 cm^−1^ corresponding to symmetric and antisymmetric stretching of C = O aliphatic group [29,30]. The FT-IR spectrum of Zn^2+^ crosslinked alginate blank beads presented bands at 1650 and 1425 cm^−1^ corresponding to the stretching of symmetric and antisymmetric carboxylic groups of the Zn-alginate complex and due to the coordination between the metal ion and the functional groups of the polymer [31]. #F5 and #F6 showed the majority of the characteristic peaks of both Zn-alginate and prednisolone, however at 1703 cm^-1^ a new band was recognized, which could be associated to the interaction between P and alginate chains *via* Zn^+2^ coordination.

### Study of release kinetics

**Fig 5:**
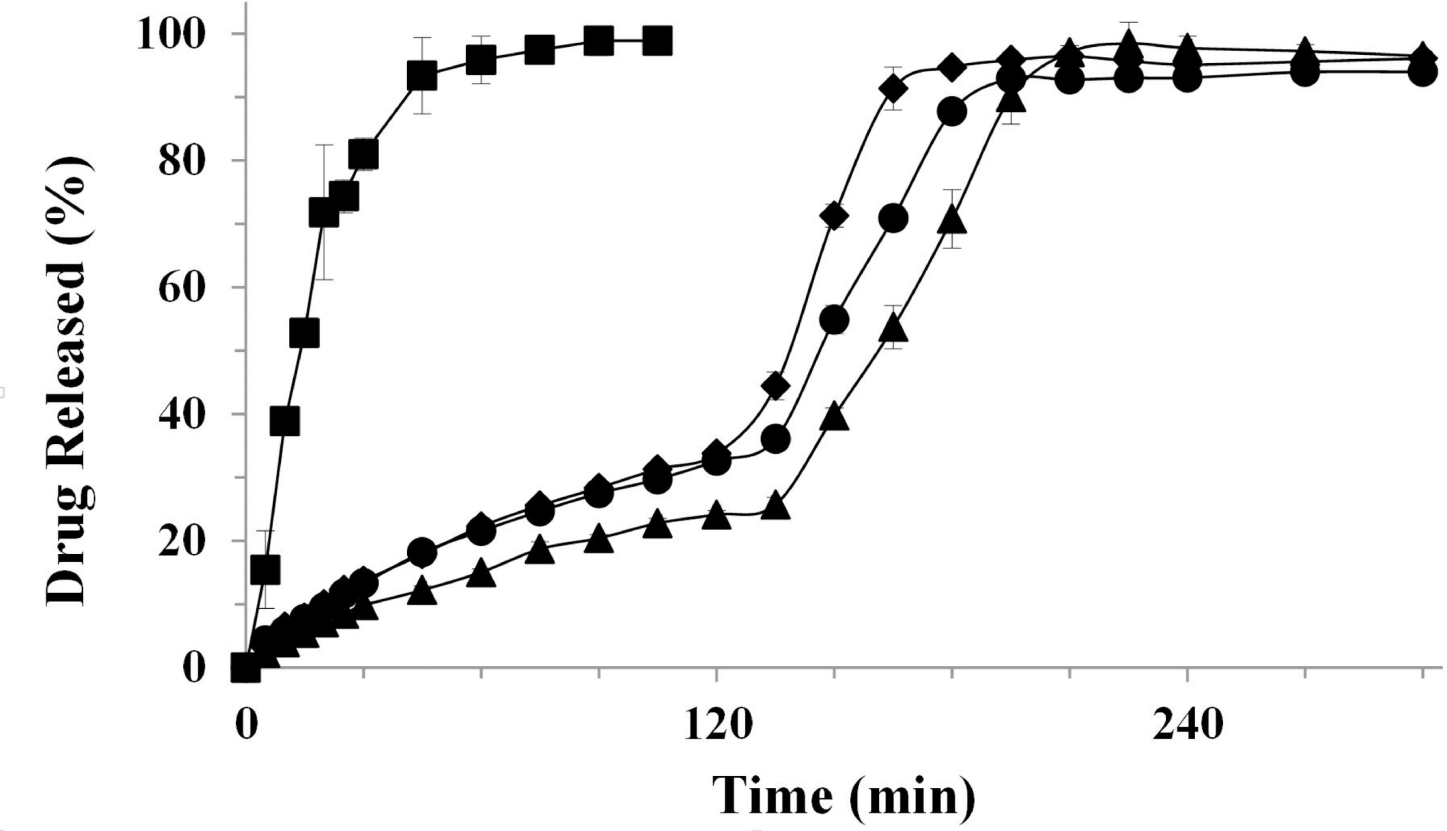
Release profiles of P from: raw material, square; # F2, diamond; # F4, circle and #F6, triangle. Mean ± SD; (n = 6).

Dissolution studies of prednisolone raw material and produced beads were performed using USP Apparatus 2 and a classic pH-change assay. The dissolution profiles of F2, F4 and F6 characterized by the highest drug/polymer ratio (1:5) are shown in Fig 5.

As expected, the dissolution profile of P raw material was typical of compound belonging to the class I of the Biopharmaceutical Classification System (high solubility and permeability trough biological membranes), the complete solubilisation was achieved in simulated gastric environment after about 90 minutes [32]. Differently, F2, F4 and F6 showed a partial gastroresistance. In particular, as shown in Fig 5, #F6 obtained with the highest alginate amount exhibited the best dissolution profile, releasing only 24.1% of the drug in simulated gastric fluid (SGF) followed after the pH change by a more sustained release in simulated intestinal fluid (SIF) with respect to #F4 and #F2 (100% of P release after about 3.5h). From the data obtained, all formulations seem to release the drug through a diffusional/erosional mechanism. However, the greatest alginate amount as in #F6 seems to be able to better retain the drug during its exposition to simulated biological fluids. Conversely in SIF (at pH 6.8) beads started to swell and further erode due to the ion-exchange process.

### In vivo studies

**Fig 6:**
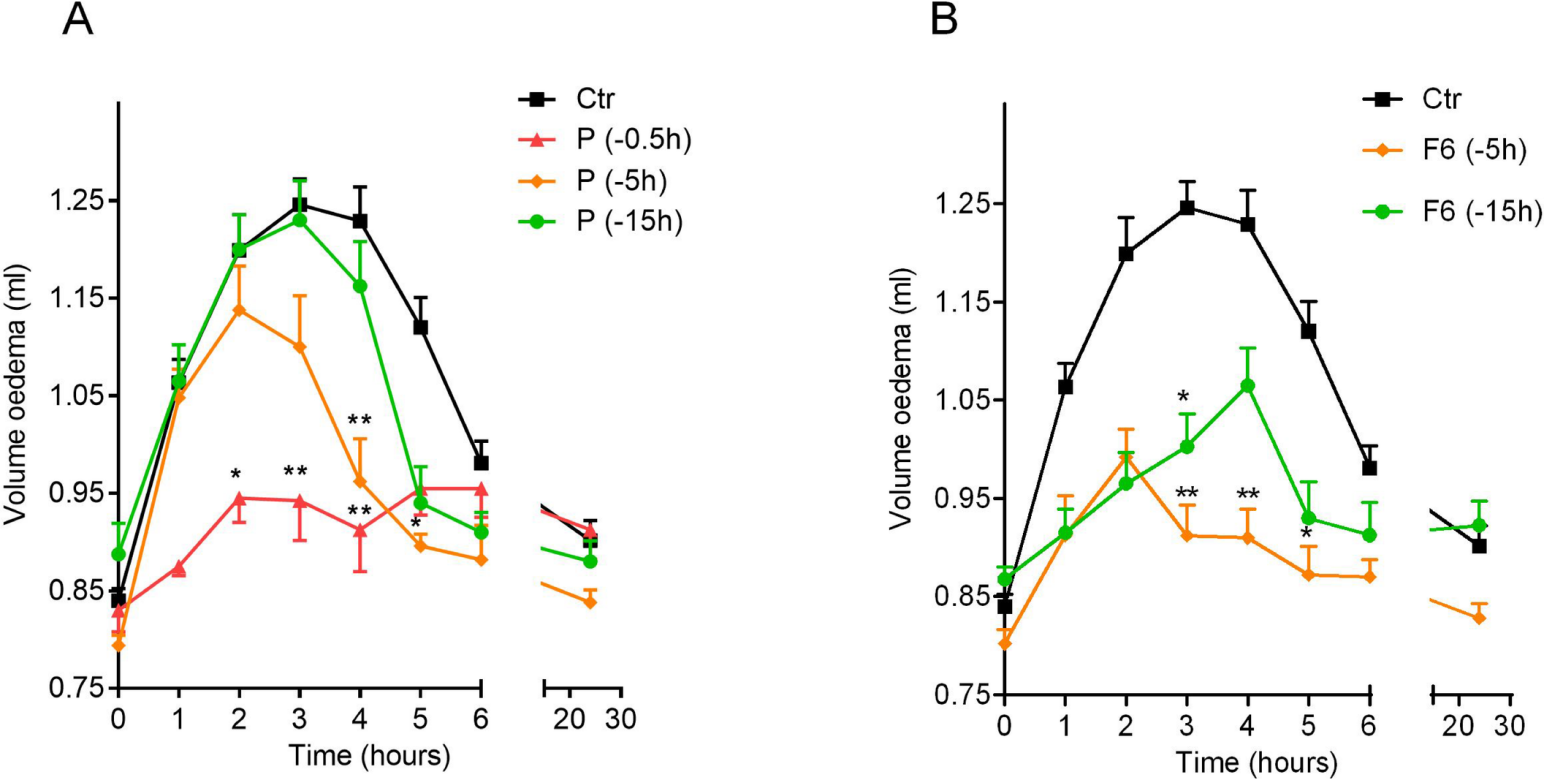
**Oedema volume reduction obtained by administering *per os* pure prednisolone (A) and #F6 (B) at different time points (15, 5, 0.5h) to rats before carrageenan injection, compared to control; mean ± SEM (*n* = 8)**. *p < 0.05, **p < 0.01 compared to control.

The anti-inflammatory activity of the formulation F6 was evaluated in rats by a carrageenan-induced oedema model and compared to the activity of pure drug. Rat paw oedema reached the maximum value (paw volume, ml) between 2 and 4 h after the injection of the phlogistic agent (Fig 6 –ctr). Preliminary experiments were performed by using three different doses of prednisolone (1–3–10 mg kg^-1^) administered by gavage to rats 0.5h before carrageenan injection (data not shown). The dose of 3 mg kg^-1^ of prednisolone was selected as it achieved the optimal anti-inflammatory effect. The pure drug (3 mg kg^-1^, prednisolone) was significantly efficacious in reducing rat paw oedema when administered at t = 0.5h before carrageenan injection (Fig 6A). The anti-inflammatory activity of prednisolone was lost when administered to rats 5h or 15 h before the phlogistic agent injection (Fig 6B).

To verify a potential delayed/prolonged *in vivo* anti-inflammatory activity, as effect of an accurate particle engineering process, the optimized formulation F6 in dose equivalent to 3 mg kg^-1^ of prednisolone was orally administered to rats 5h and 15h before carrageenan injection.

F6 significantly reduced carrageenan-induced paw oedema in rats when administered 5h before the injection of the phlogistic agent (Fig 6B). The anti-inflammatory activity of F6 still persisted when administered to rats 15h before carrageenan injection (Fig 6B), in contrast to reference pure prednisolone (3 mg kg^-1^) that was efficacious in reducing rat paw oedema only at t = 0.5h (Fig 6A). As previously reported [27], blank Zn-alginate beads (F) did not significantly affect the paw oedema when administered to rats at the same time points before oedema induction (data not shown), thus, indicating that alginate and zinc do not interfere with the inflammatory process.

### In vitro drug release from the final dosage form

Formulation #F6, acting as a partially gastro-resistant and sustained release formulation and able to exert the anti-inflammatory activity even if administered 15h before the phlogistic injury, was selected as core material for the development of a final dosage form suitable for a chronotherapy.

Therefore, #F6 was hosted into DR^®^ capsules giving F6/DR^®^ platform. DR^®^ capsules were selected, among different capsules models, for their specific feature in protecting active pharmaceutical ingredient from acidic environment thanks to the properties of a mixture of hydroxypropyl methylcellulose (HPMC) and gellan gum.

**Fig 7:**
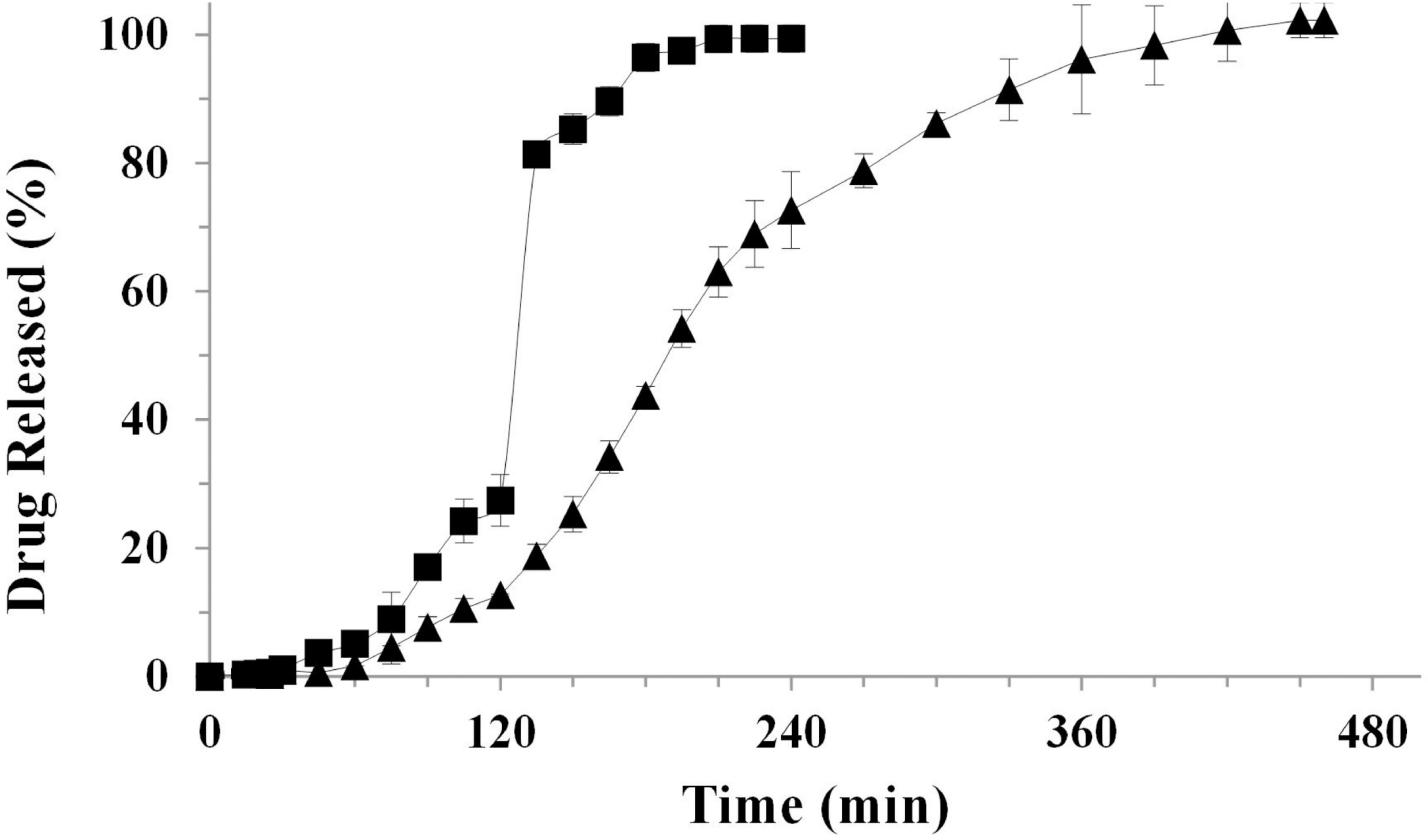
Release profiles of: P raw material hosted into DR^®^ capsules (square) compared with optimized F6/DR^®^ final dosage form (triangle). Mean ± SD; (n = 3).

The resulting dissolution profile of P from F6/DR^®^ cps versus more conventional P/DR^®^ cps profile showed that DR^®^ caps were able *per se* to delay the dissolution profile of unprocessed P (Fig 7), extending its release from 90 to 180 minutes with a significant reduction in SGF (34.2%).

Interestingly, the final dosage form F6/DR^®^ cps reduced significantly P release in SGF from 24.1% to 12.6%; at the same time, after pH change, P dissolution was significantly delayed up to about 390 minutes (6.5 hours). This optimal drug release profile was due to combination of intrinsic properties of capsules and hosted beads; only after the dissolution of the capsule body, beads are exposed to the dissolution medium, beginning later the dissolution process. Differently from gelatin capsules that normally disaggregate in simulated gastric fluid in about 5 minutes, the DR^®^ capsules begin to disaggregate after 75–90 minutes protecting the formulation from the acid environment. After this lag time, beads start to hydrate and swell, allowing a slight drug diffusion in acidic medium followed by a complete but delayed swelling and erosion processes in SIF.

## Conclusions

In this research work, we studied the effect of process and feed solution variables on particles size, morphology, solid state properties and drug release profile of prednisolone microparticles manufactured using prilling as micro-encapsulation technique and Zn-alginate as gastroresistant carrier. Particles prepared from solution containing the highest amount of polymer and drug showed good production parameters such as high encapsulation efficiency and strongly crosslinked inner matrix as evidenced by SEM pictures of cryofractured particles. Moreover, engineered particles showed a reduced P release in gastric simulated environment and a slowed release in simulated intestinal fluid, compared to conventional P.

The prolonged *in vivo* anti-inflammatory effectiveness of formulation #F6 tested by a carrageenan-induced oedema model clearly reflects its ability to release the drug in a delayed and sustained way, highlighting its potential benefits for a right chronotherapy.

Finally, the optimized formulation F6 was selected for the manufacturing of a final dosage form, using DR^®^ capsules chosen for their peculiar dissolution properties. By designing a more complex platform, hosting F6 beads in DR^®^ capsules, it was possible to further delay and prolong P release, up to 6.5 hours. The platform (#F6/ DR^®^) used in the present study, therefore, represents a suitable dosage form able to delay and extend SAID release in a time scale ideal for the successful treatment of early morning pathologies.

## References

[1] Gibbs JE, Ray DW (2013) The role of the circadian clock in rheumatoid arthritis. Arthritis Res Ther 15: 205 10.1186/ar4146 23427807 PMC3672712

[2] Straub RH, Cutolo M (2007) Circadian rhythms in rheumatoid arthritis: implications for pathophysiology and therapeutic management. Arthritis Rheum 56: 399–408. 17265475 10.1002/art.22368

[3] Bijlsma JW, Jacobs JW (2008) Glucocorticoid chronotherapy in rheumatoid arthritis. Lancet 371: 183–184. 10.1016/S0140-6736(08)60114-2 18207003

[4] Spies CM, Straub RH, Cutolo M, Buttgereit F (2014) Circadian rhythms in rheumatology—a glucocorticoid perspective. Arthritis Res Ther 16 Suppl 2: S3. 10.1186/ar4687 PMC4249493 25608777

[5] Haus E, Sackett-Lundeen L, Smolensky MH (2012) Rheumatoid arthritis: circadian rhythms in disease activity, signs and symptoms, and rationale for chronotherapy with corticosteroids and other medications. Bull NYU Hosp Jt Dis 70 Suppl 1: 3–10. 23259651

[6] Buttgereit F, Doering G, Schaeffler A, Witte S, Sierakowski S, Gromnica-Ihle E, et al (2008) Efficacy of modified-release versus standard prednisone to reduce duration of morning stiffness of the joints in rheumatoid arthritis (CAPRA-1): a double-blind, randomised controlled trial. Lancet 371: 205–214. 10.1016/S0140-6736(08)60132-4 18207016

[7] Alten R, Grahn A, Holt RJ, Rice P, Buttgereit F (2015) Delayed-release prednisone improves fatigue and health-related quality of life: findings from the CAPRA-2 double-blind randomised study in rheumatoid arthritis. RMD Open 1: e000134 10.1136/rmdopen-2015-000134 26535146 PMC4623361

[8] Alten R, Holt R, Grahn A, Rice P, Kent J, Buttgereit F, et al (2015) Morning stiffness response with delayed-release prednisone after ineffective course of immediate-release prednisone. Scand J Rheumatol 44: 354–358. 10.3109/03009742.2015.1038582 26114379 PMC4732433

[9] Buttgereit F, Kent JD, Holt RJ, Grahn AY, Rice P, Alten R, et al (2015) Improvement Thresholds for Morning Stiffness Duration in Patients Receiving Delayed- Versus Immediate-Release Prednisone for Rheumatoid Arthritis. Bull Hosp Jt Dis (2013) 73: 168–177. 26535595

[10] Davis M, Williams R, Chakraborty J, English J, Marks V, Ideo G, et al (1978) Prednisone or prednisolone for the treatment of chronic active hepatitis? A comparison of plasma availability. Br J Clin Pharmacol 5: 501–505. 656293 10.1111/j.1365-2125.1978.tb01664.x PMC1429358

[11] Pickup ME (1979) Clinical pharmacokinetics of prednisone and prednisolone. Clin Pharmacokinet 4: 111–128. 378499 10.2165/00003088-197904020-00004

[12] Madsbad S, Bjerregaard B, Henriksen JH, Juhl E, Kehlet H (1980) Impaired conversion of prednisone to prednisolone in patients with liver cirrhosis. Gut 21: 52–56. 7364321 10.1136/gut.21.1.52 PMC1419565

[13] Di Colo G, Baggiani A, Zambito Y, Mollica G, Geppi M, Serafini MF (2006) A new hydrogel for the extended and complete prednisolone release in the GI tract. Int J Pharm 310: 154–161. 16414222 10.1016/j.ijpharm.2005.12.002

[14] Lau ET, Johnson SK, Mikkelsen D, Halley PJ, Steadman KJ (2012) Preparation and in vitro release of zein microparticles loaded with prednisolone for oral delivery. J Microencapsul 29: 706–712. 10.3109/02652048.2012.686527 22612552

[15] Okimoto K, Miyake M, Ohnishi N, Rajewski RA, Stella VJ, Irie T, et al (1998) Design and evaluation of an osmotic pump tablet (OPT) for prednisolone, a poorly water soluble drug, using (SBE)7m-beta-CD. Pharm Res 15: 1562–1568. 9794499 10.1023/a:1011955117026

[16] Skowyra J, Pietrzak K, Alhnan MA (2015) Fabrication of extended-release patient-tailored prednisolone tablets via fused deposition modelling (FDM) 3D printing. Eur J Pharm Sci 68: 11–17. 10.1016/j.ejps.2014.11.009 25460545

[17] Auriemma G, Mencherini T, Russo P, Stigliani M, Aquino RP, Del Gaudio P (2013) Prilling for the development of multi-particulate colon drug delivery systems: pectin vs. pectin-alginate beads. Carbohydr Polym 92: 367–373. 10.1016/j.carbpol.2012.09.056 23218307

[18] Auriemma G, Del Gaudio P, Barba AA, d'Amore M, Aquino RP (2011) A combined technique based on prilling and microwave assisted treatments for the production of ketoprofen controlled release dosage forms. Int J Pharm 415: 196–205. 10.1016/j.ijpharm.2011.05.077 21679754

[19] Del Gaudio P, Colombo P, Colombo G, Russo P, Sonvico F (2005) Mechanisms of formation and disintegration of alginate beads obtained by prilling. Int J Pharm 302: 1–9. 16102925 10.1016/j.ijpharm.2005.05.041

[20] Almeida-Prieto S, Blanco-Mendez J, Otero-Espinar FJ (2004) Image analysis of the shape of granulated powder grains. J Pharm Sci 93: 621–634. 14762901 10.1002/jps.10572

[21] Cerciello A, Auriemma G, Del Gaudio P, Cantarini M, Aquino RP (2016) Natural polysaccharides platforms for oral controlled release of ketoprofen lysine salt. Drug Dev Ind Pharm 30: 1–19. 10.1080/03639045.2016.1195401 27237337

[22] Cicala C, Morello S, Alfieri A, Vellecco V, Marzocco S, Autore G (2007) Haemostatic imbalance following carrageenan-induced rat paw oedema. Eur J Pharmacol 577: 156–161. 17850787 10.1016/j.ejphar.2007.08.007

[23] Chakraborty S, Khandai M, Sharma A, Khanam N, Patra Ch N, Dinda SC, et al (2010) Preparation, in vitro and in vivo evaluation of algino-pectinate bioadhesive microspheres: An investigation of the effects of polymers using multiple comparison analysis. Acta Pharm 60: 255–266. 10.2478/v10007-010-0026-7 21134861

[24] Chakraborty S, Khandai M, Sharma A, Khanam N, Patra C, Dinda S, et al (2010) Preparation, in vitro and in vivo evaluation of algino-pectinate bioadhesive microspheres: An investigation of the effects of polymers using multiple comparison analysis. Acta pharmaceutica 60: 255–266. 10.2478/v10007-010-0026-7 21134861

[25] Del Gaudio P, Auriemma G, Russo P, Mencherini T, Campiglia P, Stigliani M, et al (2014) Novel co-axial prilling technique for the development of core-shell particles as delayed drug delivery systems. Eur J Pharm Biopharm 87: 541–547. 10.1016/j.ejpb.2014.02.010 24582614

[26] Vogt M, Derendorf H, Kramer J, Junginger HE, Midha KK, Shah VP, et al (2007) Biowaiver monographs for immediate release solid oral dosage forms: prednisolone. J Pharm Sci 96: 27–37. 17039494 10.1002/jps.20768

[27] Cerciello A, Auriemma G, Morello S, Pinto A, Del Gaudio P, Russo P, et al (2015) Design and In Vivo Anti-Inflammatory Effect of Ketoprofen Delayed Delivery Systems. J Pharm Sci 104: 3451–3458. 10.1002/jps.24554 26088065

[28] Cerciello A, Auriemma G, Del Gaudio P, Sansone F, Aquino RP, Russo P (2016) A novel core-shell chronotherapeutic system for the oral administration of ketoprofen Journal of Drug Delivery Science and Technology 32, Part B: 126–131.

[29] Palanisamy M, Khanam J (2011) Solid dispersion of prednisolone: solid state characterization and improvement of dissolution profile. Drug development and industrial pharmacy 37: 373–386. 10.3109/03639045.2010.513984 20839923

[30] Mazurek S, Szostak R (2012) Quantitative Determination of Prednisone in Tablets by Infrared Attenuated Total Reflection and Raman Spectroscopy. Journal of AOAC International 95: 744–750. 22816265 10.5740/jaoacint.sge_mazurek

[31] Papageorgiou SK, Kouvelos EP, Favvas EP, Sapalidis AA, Romanos GE, Katsaros FK (2010) Metal–carboxylate interactions in metal–alginate complexes studied with FTIR spectroscopy. Carbohydrate Research 345: 469–473. 10.1016/j.carres.2009.12.010 20044077

[32] Sugawara S, Imai T, Otagiri M (1994) The controlled release of prednisolone using alginate gel. Pharm Res 11: 272–277. 8165187 10.1023/a:1018963626248

